# An Unusual Case of Fibrous Dysplasia, Temporomandibular Joint Ankylosis, and Eagle’s Syndrome

**DOI:** 10.7759/cureus.68172

**Published:** 2024-08-30

**Authors:** Gurmehr Singh, Senthil Murugan P, Gheena S, Manishaa V

**Affiliations:** 1 Department of Oral and Maxillofacial Surgery, Saveetha Dental College and Hospital, Saveetha Institute of Medical and Technical Sciences, Saveetha University, Chennai, IND; 2 Department of Oral and Maxillofacial Pathology and Microbiology, Saveetha Dental College and Hospital, Saveetha Institute of Medical and Technical Sciences, Saveetha University, Chennai, IND

**Keywords:** patient specific implant (psi), mandible reconstruction, innovative technique, fibrous dysplasia (fd), tmj surgical cases

## Abstract

Fibrous dysplasia is a benign bone disease in children and young adults. This is characterized by the replacement of normal bone with fibrous tissue along with immature woven bone. Fibrous dysplasia is a rare disorder and has variable presentations that pose challenges in diagnosis and treatment. Decisions are made on a case-by-case basis, depending on the symptoms, location, or possible complications. Symptomatic lesions are treated with surgical resection. cosmetic concerns of the patients are taken care of by surgical contouring. For any unresectable or recurrent lesion, bisphosphonate therapy can be used as a form of medical management.

## Introduction

Fibrous dysplasia is a rare benign bone disorder of unknown cause, in which normal bone structure is replaced by fibrous tissue and immature woven trabecular bone formation. It can be monostotic, involving a single bone, or polyostotic, involving multiple bones [1]. Symptoms depend on the site involved and the amount of damage done, but usually involve pain and swelling in the area involved and reduced function [2]. Diagnosis is usually achieved radiographically and by histopathological examination. The treatments are triaged to observation, surgery (contouring and resection), and medical management with bisphosphonates [3]. This case report talks about a 31-year-old female who presented with monostotic fibrous dysplasia of the right-side ramus of the mandible along with ankylosis temporomandibular joint (TMJ) on the same side and an elongated right styloid process. Her chief complaint was right-side facial pain and swelling for the last four years. Plain radiographs demonstrated a mixed lytic and sclerotic lesion compatible with fibrous dysplasia. The diagnosis was finally confirmed by histopathological examination. The surgical resection of the lesion was done with significant improvement in symptoms and cosmetic concerns. We had regular follow-up visits to monitor recurrence. This case illustrates the clinical features of fibrous dysplasia and diagnostic modalities, as well as the interventions to be undertaken in order to provide optimal care for such patients.

## Case presentation

A 31-year-old female presented to the Department of Oral and Maxillofacial Surgery, at Saveetha Dental College, Chennai, with the chief complaint of gradually increasing facial pain and swelling over the past four years. She reported no history of trauma to the face or recent dental procedures. Pain was described as dull and aching, localized to the right side of her face. No history of surgery or significant medical conditions were reported. She denied any family history of bone disorders or other genetic conditions.

**Figure 1:**
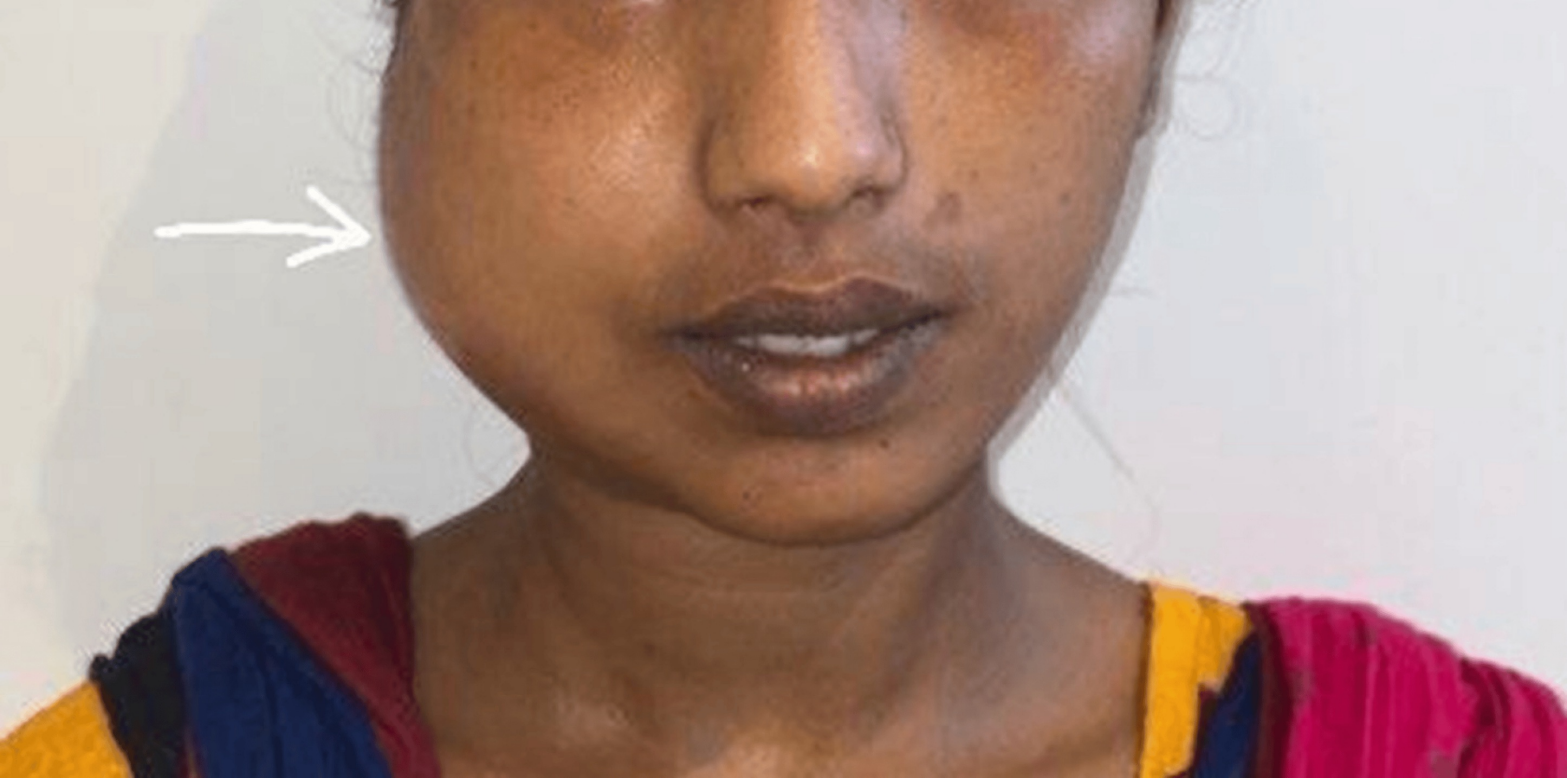
Pre-operative photograph

Upon physical examination, the patient appeared well-nourished and in no acute distress (Figure 1). Swelling along with asymmetry was noted on the right side of the face, involving the ramus of the mandible. The swelling was poorly defined and bony-hard on palpation. There was no erythema or warmth on palpation. No evidence of dental caries or periodontal disease was noted. The mouth opening was 15 mm.

**Figure 2:**
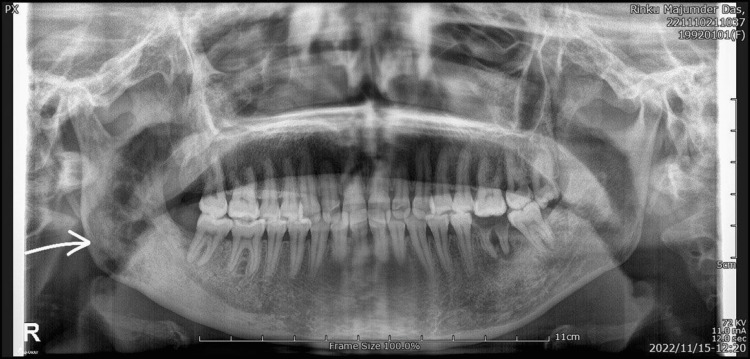
Pre-operative orthopantomogram showing bony changes in the ramus on the right side of the mandible

**Figure 3:**
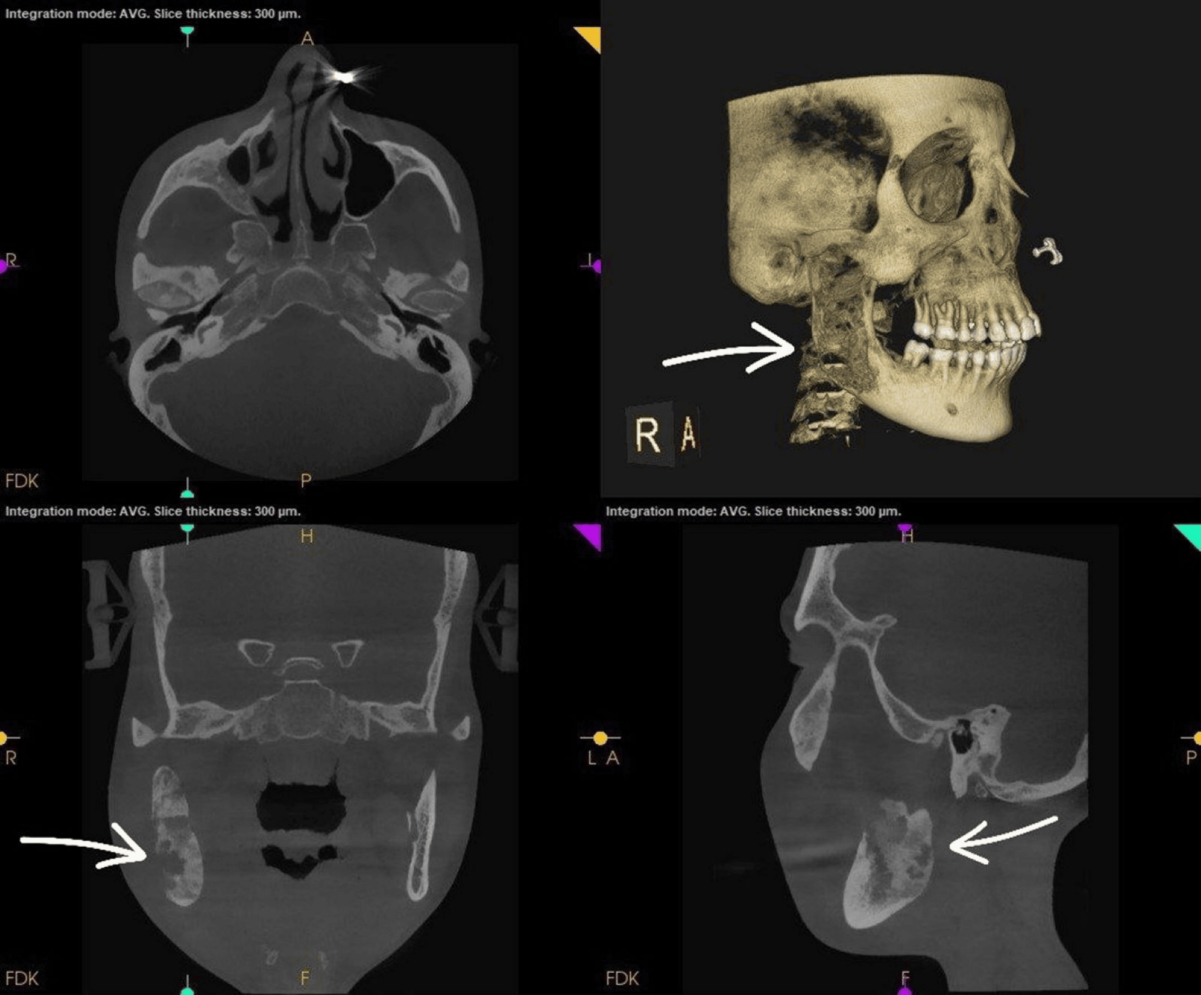
Pre-operative CT scan showing bony changes in the ramus on the right side of the mandible CT - Computed Tomography

Radiological examination of the face revealed mixed lytic as well as sclerotic lesions involving the right ramus of the mandible, consistent with fibrous dysplasia (Figures 2, 3).

**Figure 4:**
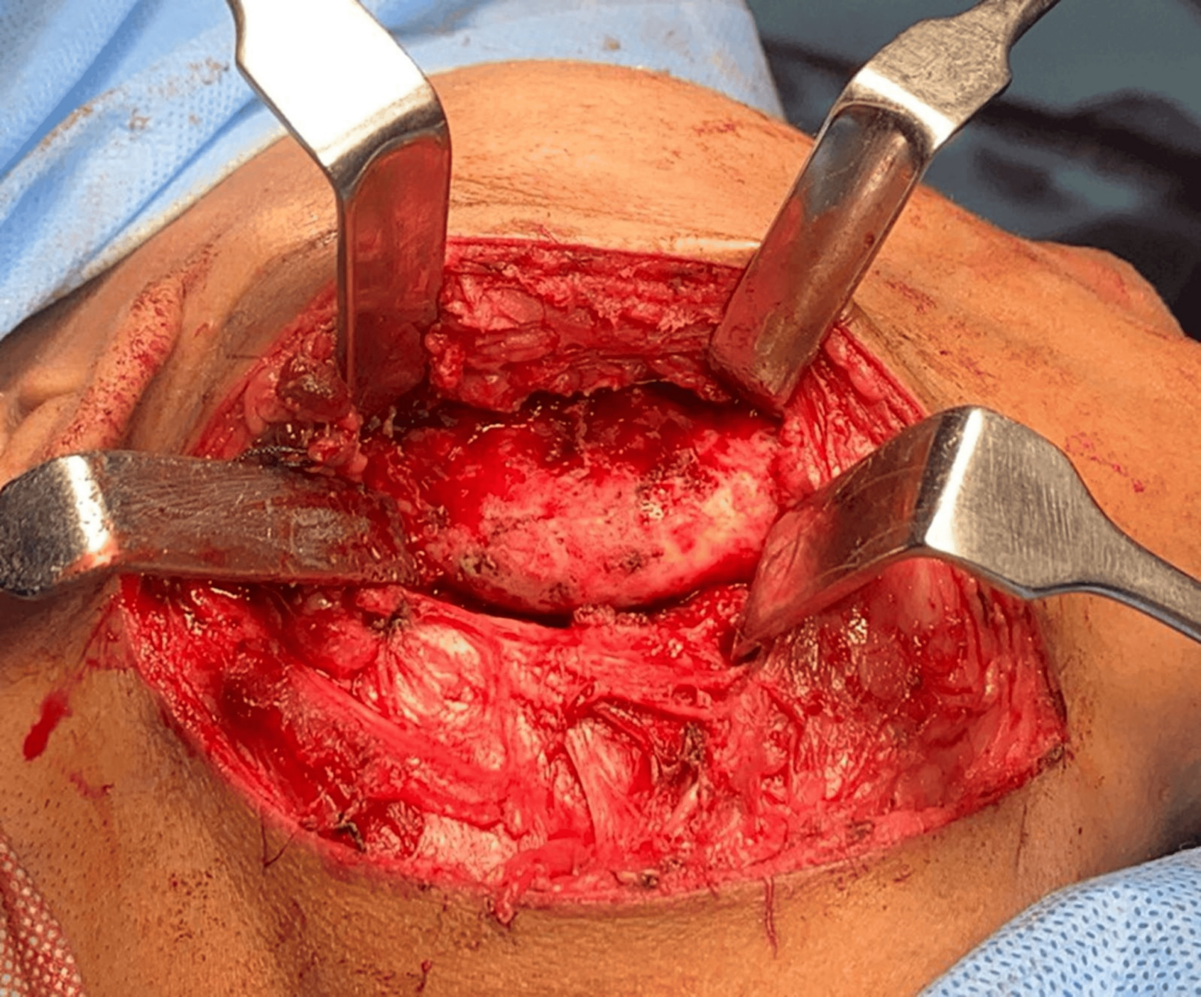
Surgical site exposed and resection carried out

**Figure 5:**
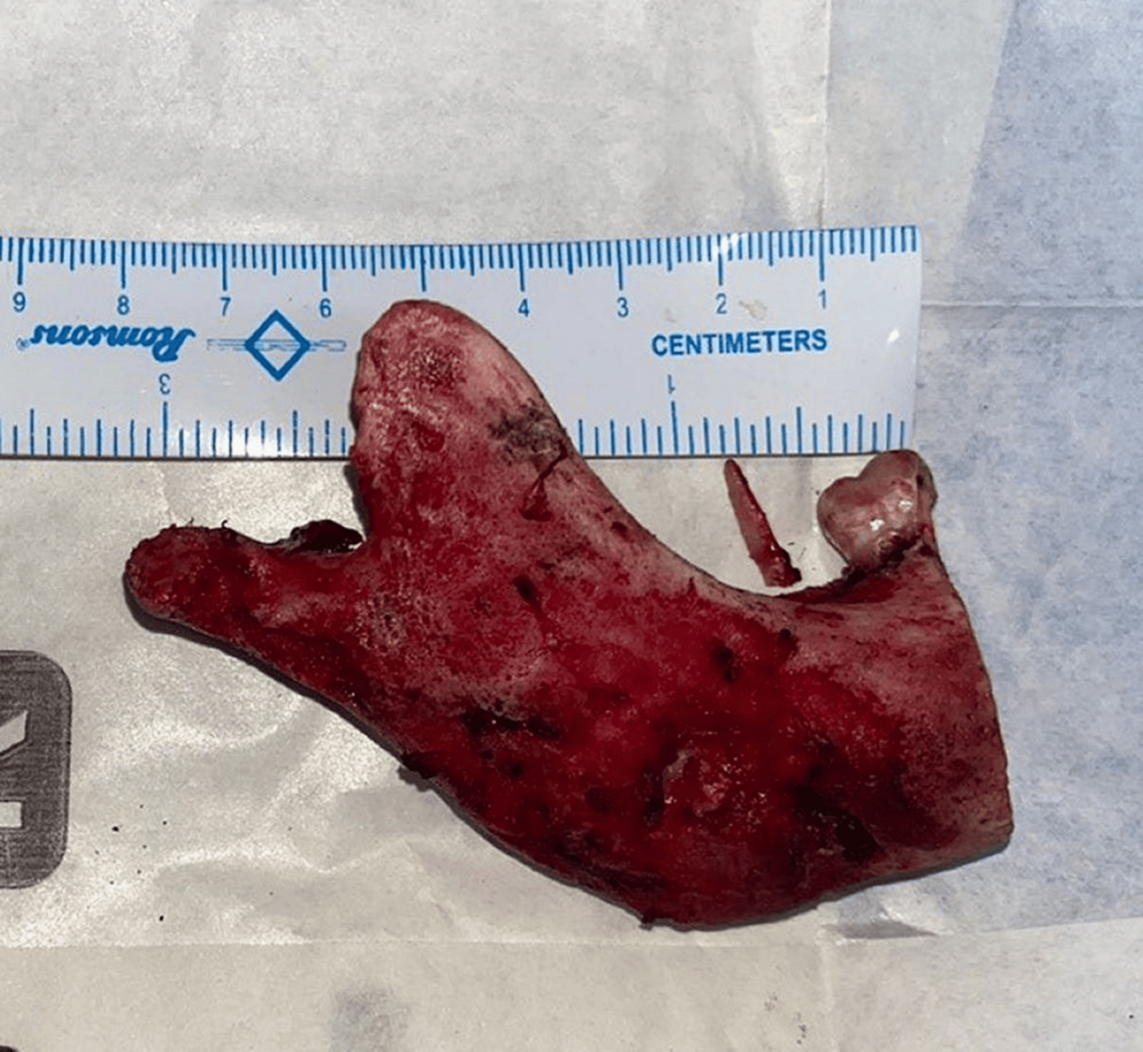
Resected specimen

**Figure 6:**
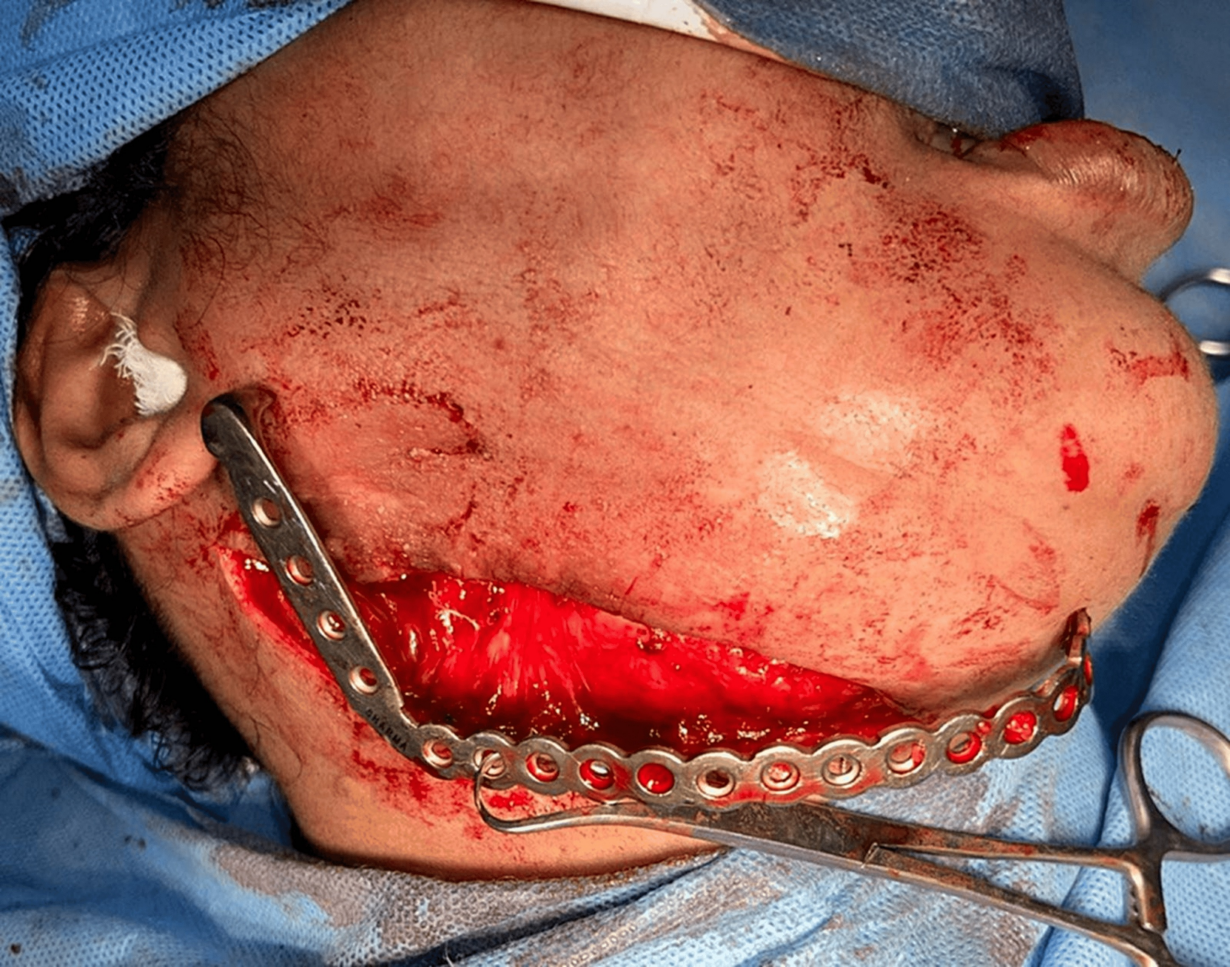
Fixation of reconstruction plate

Incisional biopsy of the lesion confirmed the presence of fibrous tissue with scattered trabeculae of woven bone. The patient was diagnosed with monostotic fibrous dysplasia of the right ramus of the mandible along with right TMJ ankylosis and Eagle's syndrome. Treatment options, about which that had been told, were observation, surgical resection followed by reconstruction, or medical management with bisphosphonates, depending on the size and the symptoms of the lesion. As the patient was not comfortable with the lesion on her face, the continuous pain, and the cosmetic deformity that it had caused her, she opted for the surgical option of resection of the lesion. Wide local excision with segmental mandibulectomy with styloidectomy and reconstruction plate fixation under general anesthesia. In December 2022, the patient underwent the procedure. Under general anesthesia, naso-endotracheal intubation was done. Standard scrubbing and draping were done. Submandibular incision and intra-oral vestibular incision were given (Figure 4). Resection was carried out distal to tooth number 46, including the condyle and coronoid process (Figure 5). The styloid process was reduced. This was followed by reconstruction plate fixation. The closure was done via 3-0 Vicryl after achieving hemostasis (Figure 6).

**Figure 7:**
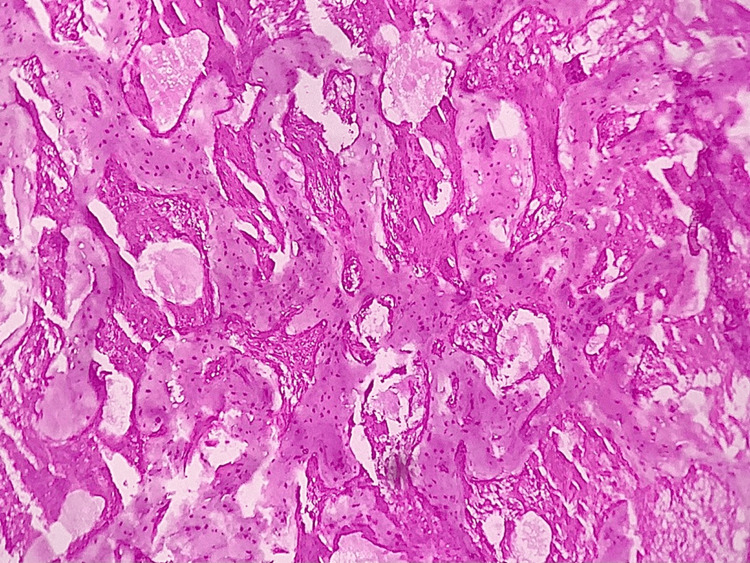
Histopathological examination of the specimen confirming the diagnosis of fibrous dysplasia

Histopathological examination of the resected specimen was consistent with the diagnosis (Figure 7). The section showed irregular curvilinear and interconnecting trabeculae of lamellar bone with lacunae composed of osteocytes in a connective tissue stroma. There was evidence of separation of the bone from the adjacent stroma suggestive of peri trabecular clefting. The intervening stroma showed minimal inflammatory cell infiltration and areas of hemorrhage. Biopsy confirmed the diagnosis of fibrous dysplasia.

**Figure 8:**
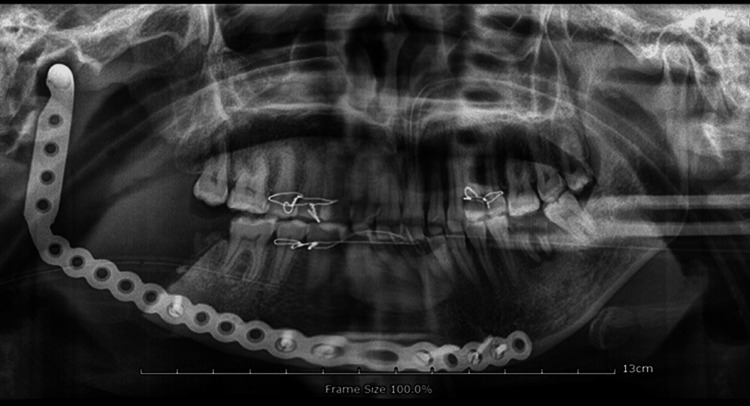
Post-operative OPG showing the fixed reconstruction plate OPG - Orthopantomogram

Postoperatively, the patient noted a marked improvement in her facial pain and swelling and she was scheduled for regular follow-up. This follow-up period included radiological examinations like taking an orthopantomogram (Figure 8).

**Figure 9:**
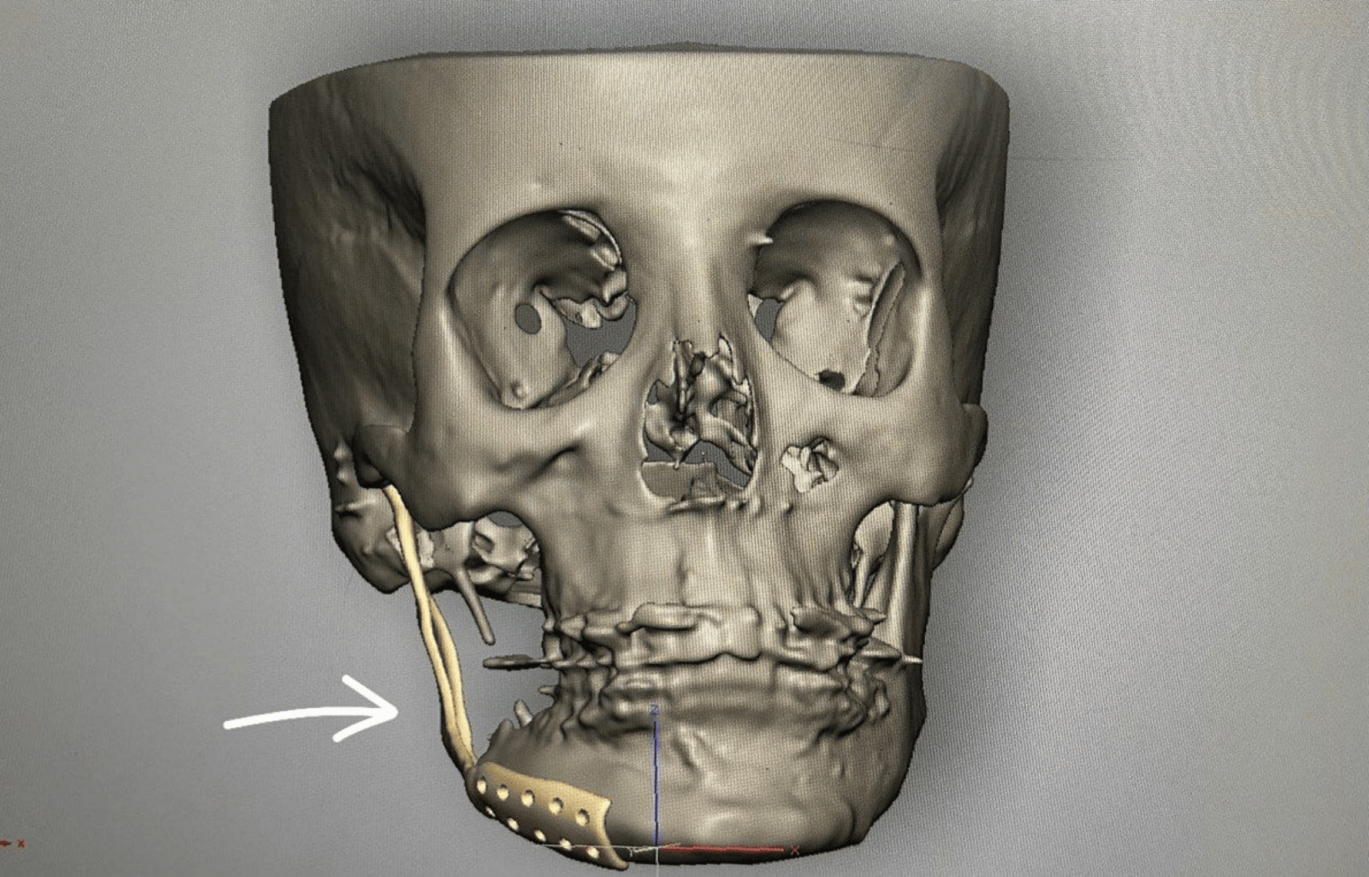
Planning and PSI designing for the reconstruction of the resected mandible PSI - Patient-specific implant

After more than one year of regular follow-up, it was decided to perform computer-aided reconstruction with a titanium-based patient-specific implant (PSI) of her defect, since there was an infection and hardware failure. Virtual designing of the implant was done and finalized on the STL model (Figure 9).

**Figure 10:**
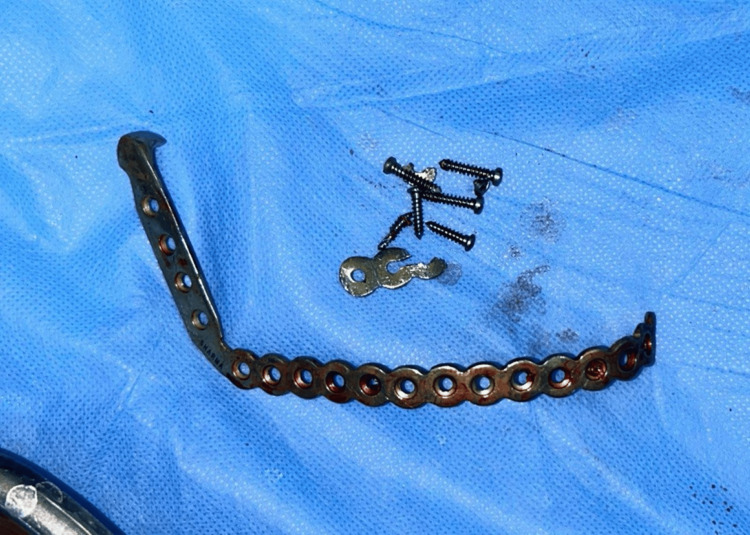
Fixation material removed

**Figure 11:**
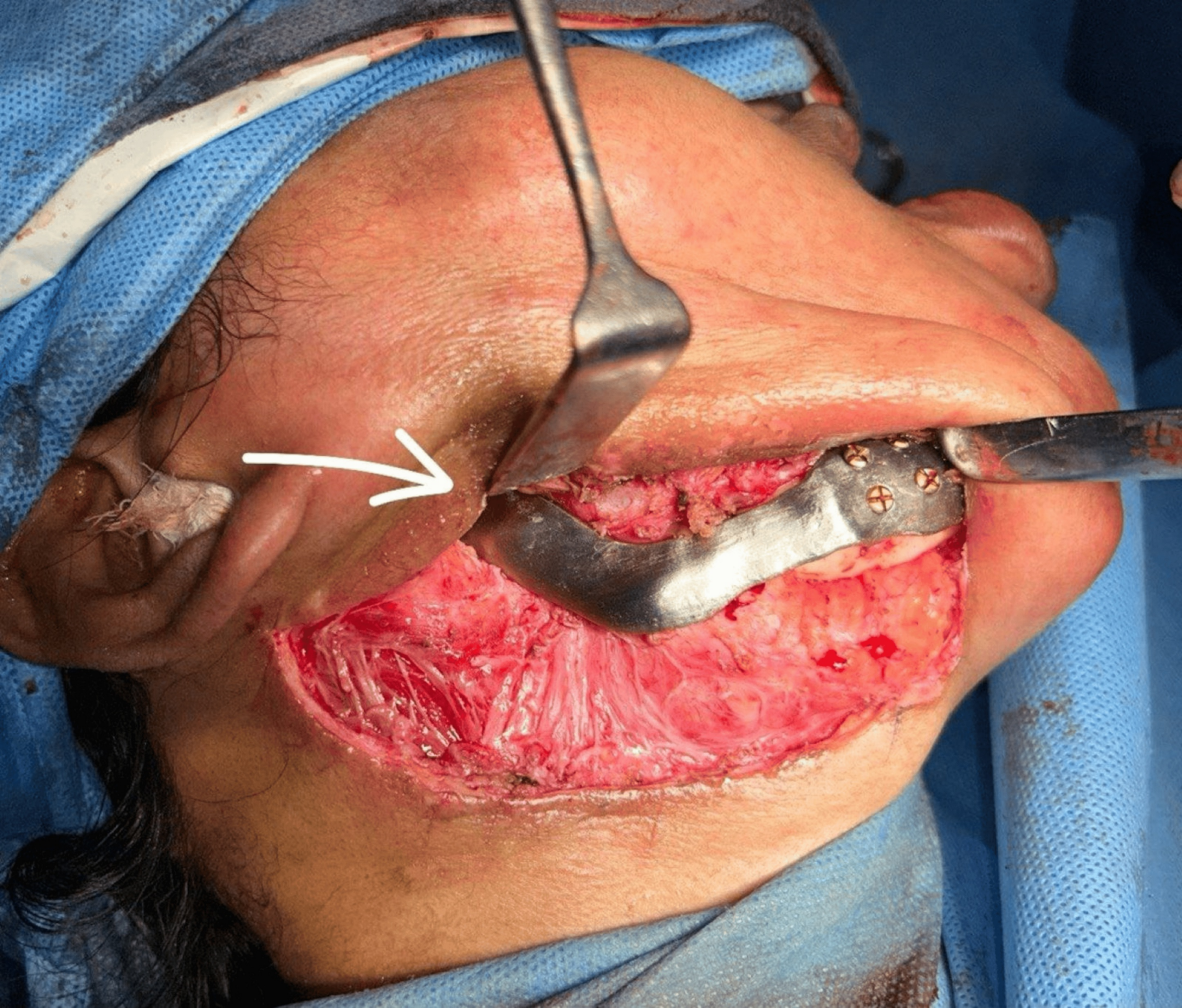
Placement of the patient-specific implant

**Figure 12:**
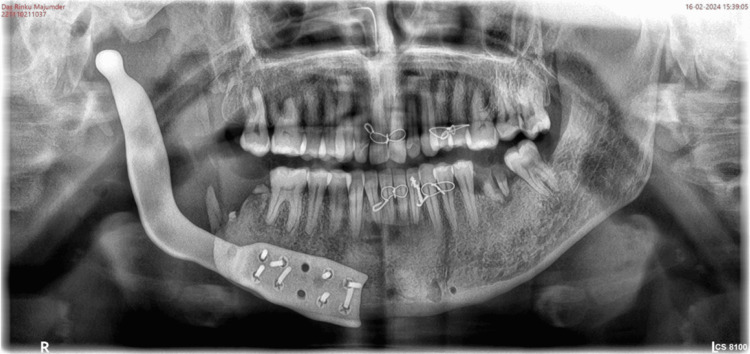
Post-operative OPG showing the successful placement of the PSI OPG - Orthopantomogram, PSI - Patient-specific implant

In February 2024, the patient underwent the procedure. Under general anesthesia, naso-endotracheal intubation was done. Standard scrubbing and draping were done. Extra-oral incision was given from an already present scar and intra-oral vestibular incision was given. A mucoperiosteal flap was raised. The reconstruction plate was removed, and PSI was placed (Figures 10, 11). A tight primary closure was given intra-orally and extra-orally was given after achieving hemostasis. The position of the PSI was confirmed by taking an Orthopantomogram post-operatively (Figure 12).

**Figure 13:**
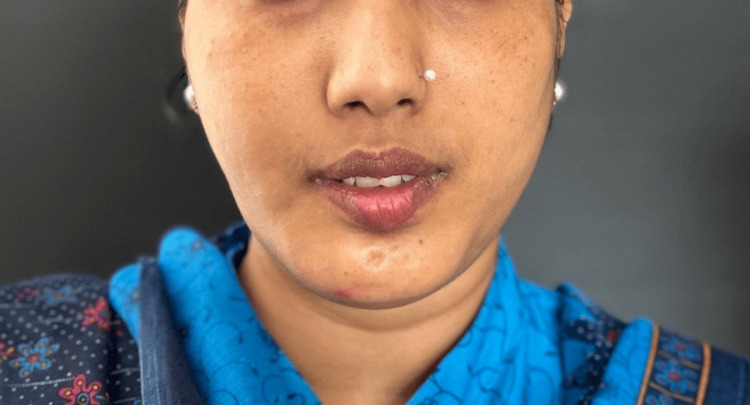
Post-operative photograph taken after 30 days of the surgery

The patient has been now on regular follow-up and is satisfied with the treatment and her esthetics (Figure 13).

## Discussion

Upon clinical and radiological examination, we can mistake fibrous dysplasia with a number of conditions. Paget's disease is a bone disorder characterized by abnormal bone remodeling, leading to enlarged and weakened bones. It can affect the skull and facial bones, leading to deformities that may resemble fibrous dysplasia. Cherubism is a rare genetic disorder that leads to bilateral swelling of the upper and lower jaws, giving the patients a characteristic “cherubic” appearance. It differs from fibrous dysplasia based on its distinct radiographic features and genetic testing. Ossifying fibroma is a benign bone tumor that can affect the craniofacial bones. It may present with radiographic features similar to fibrous dysplasia, but the histopathological features can help differentiate between the two conditions. Osteosarcoma is a malignant bone tumor that can occur in the craniofacial region. It can sometimes be mistaken for fibrous dysplasia radiographically, but a biopsy is necessary to confirm the diagnosis. Simple bone cysts are fluid-filled cavities that can occur in the bones, including the craniofacial bones. They may present with similar radiographic features as fibrous dysplasia but can be differentiated based on their distinct appearance in imaging studies.

Fibrous dysplasia is a rare bone disease, which is a type of polyostotic or monostotic. It is a benign disease that affects bones and develops mainly in adolescents and young people. In this, normal structure is replaced by fibrous tissue, creating woven bone. The disease may involve only one bone, or multiple bones, which will be categorized as polyostotic [4]. Frequent testing for Fibrous dysplasia includes radiographic imaging of affected bones and histopathological examination. Treatment options are surgery contouring, surgical resection, and pharmacological treatment with bisphosphonates [5]. The disorder is occasionally asymptomatic and usually not life-threatening. Most of the concerns are in relation to the form and function of the affected part. Fibrous dysplasia is caused by a genetic mutation that occurs in early fetal development [6]. The mutation is in the Guanine nucleotide-binding protein, alpha-stimulating (*GNAS*) gene, which gives the body instructions to make a protein that helps facilitate the activity of cells [7].

The mutation responsible for fibrous dysplasia is typically not inherited from a patient’s parents but occurs randomly during the formation of the ovum or sperm cells. This is known as a somatic mutation, meaning it is present only in certain cells of the body and is not passed on to offspring [8]. The mutation in the *GNAS *gene leads to the production of an altered form of the G protein, which plays a role in transmitting signals from outside the cell to the cell's interior [9]. This changed protein disrupts the natural development and remodeling of bone and leads to the creation of abnormal fibrous tissue instead of bone. These tissues can develop randomly, hence the sporadic type of fibrous dysplasia. However, fibrous dysplasia can also be related to a bigger genetic condition called McCune-Albright syndrome. In this, *GNAS* gene mutation is present in a very high proportion of the body's cells leading to a more severe and widespread form of fibrous dysplasia. In addition to these symptoms, the person may also exhibit changes to their skin pigmentation and variations in hormonal disturbances [10]. The specific treatment for fibrous dysplasia varies by the nature and seriousness of each person's symptoms, including whether one or more bones are involved; to restore function, pain management, and reduce fracture incidence. Treatment of fibrous dysplasia depends on the location, extent of involvement, and symptoms along with overall health status [11].

Common treatment options employed for patients with fibrous dysplasia include observation, medications, and surgery. In cases where fibrous dysplasia is not causing any symptoms or functional impairment, a “watch and wait” approach may be taken. Regular monitoring with imaging studies like X-rays, CT scans, or MRIs may be recommended to track any changes in bone over time. Some of the medicines like bisphosphonates might be prescribed in a few cases to help in strengthening bone and also to decrease the risk of any fractures. Surgery may be considered for symptomatic cases of fibrous dysplasia that are causing pain, deformity, or functional impairment. The goals of surgery may include relieving pain, improving function, correcting deformities as well as reducing the risk of fractures. Surgical options can include pain, improving function, correcting deformities as well as reducing the risk of fractures [12]. Surgical options can include curettage (this involves scraping out abnormal tissue and filling space with bone graft or other materials), contouring and resection, bone grafting (adding bone tissue to repair or strengthen affected bone), internal fixation (using screws, plates, or rods to stabilize the bone) and joint replacement (in cases where fibrous dysplasia affects a joint, joint replacement surgery may be considered). Surgical resection and contouring involve cutting along with repositioning bone to correct deformities [13]. Regardless of the treatment approach taken, regular follow-up with the healthcare provider is essential to monitor the progression of the disease, manage symptoms along address any complications that may arise [14].

Fibrous dysplasia is a disorder associated with numerous difficulties regarding its diagnosis and management. The former is a result of the disease's rarity and varied clinical implications. The latter, in turn, should be based on the symptoms at hand, including the affected location or possible complications. In most cases, surgical resection is the treatment of choice for symptomatic lesions. However, in cases where the lesion is unresectable or the disease keeps recurring, medical management with bisphosphonates is an alternative [15]. In the case discussed here, the patient was followed up for one year, and reconstruction was planned which included PSI placement. PSIs are custom-made implants that are particularly designed for that patient based on the patient's own anatomy. These implants are manufactured using a combination of a CT or an MRI scan of the area needing the PSI, anatomical molding and shaping to create a model of the implant, and finally improved manufacturing. PSIs are increasingly becoming significant in different medical antibodies, such as maxillofacial surgery, where they can be used to fix and repair complex anatomical defects and injuries. Reconstruction of the mandible can be a cumbersome process, as it is essential to perform sufficient planning and execution with a high level of precision to restore the function and aesthetics of the jaw. PSIs have changed the approach to mandibular reconstruction for all time, as they offer custom alternatives and solutions that securely fit patient's mandibles [16].

Before the surgery, the patient undergoes a CT scan or MRI scan. These images are used to create a 3D model of a patient's anatomy, allowing the surgeons to visualize defects and plan reconstruction procedures accurately. Using the 3D model, the surgeon collaborates with engineers and designers (and prosthodontists in case it involves a dental segment) to create a custom implant that precisely fits the defect. Design is tailored to the patient's specific anatomy, ensuring optimal fit and function. Once the design is finalized, the implant is manufactured using CAM Technologies (Nottingham, MD). The implant is typically made from biocompatible materials such as titanium or medical-grade polymers. During surgery, the surgeon removes the diseased or damaged portion of the maxilla or mandible and prepares the site for implantation. The customized PSI is then placed in the defect site, ensuring perfect fit and alignment with the remaining mandible.

Benefits of PSIs in mandibular reconstruction include precision, reduced surgery time, improved esthetics, and faster recovery. PSIs offer a high degree of precision and accuracy in fitting a patient's anatomy, reducing the risk of complications as well as improving surgical outcomes. Custom implants can streamline surgical procedures by eliminating the need for intraoperative adjustments, leading to shorter operative times along with reduced anesthesia exposure. PSIs can help restore the natural shape and contour of the mandible, improving facial symmetry and aesthetics for patients. By reducing surgical complexity along with improving the fit, PSIs can potentially lead to faster recovery times and reduced postoperative discomfort for patients [17]. Patient-specific implants are an essential part of the mandible's reconstruction process. Personalized solutions help to increase the level of precision and hence, the outcome of the surgery performed on the patient. Therefore, it can be seen as an important progress in the field of maxillofacial surgery as it allows analyzing the peculiarity of every patient's anatomy and provides a solution suitable for it. The success rate of PSIs for the mandible's reconstruction varies depending on the count of factors. Everything starts with the diagnosis and the choice of treatment. Thus, PSI is only a part of the treatment, and the outcomes are also highly dependent on the patient's condition and the surgery itself, as well as the quality of the implant. However, by providing a higher level of precision and quality for medical devices, the efficiency of such implants can be seen as high [18]. Thorough preoperative planning, including detailed imaging along with the precise design of the implant, is crucial for the success of PSIs in mandibular reconstruction. Accurate planning helps ensure that the implant will integrate seamlessly with the patient's existing anatomy.

Skill and experience of the surgical team performing procedures play significant roles in the success of mandibular PSIs. Proper surgical technique, including precise placement of the implant and meticulous attention to detail during the procedure, is essential for optimal outcomes. The choice of materials used to manufacture an implant can impact its success rate. Biocompatible materials such as titanium or medical-grade polymers are commonly used for PSIs to minimize the risk of adverse reactions or implant rejection. Adequate postoperative care and follow-up are essential for monitoring the patient's recovery along with ensuring the long-term success of the implant. Close monitoring of healing, function, and potential complications can help address any issues promptly and optimize outcomes [19].

It is more than possible to conclude that patient-specific implants for mandible reconstruction display promising success rates when patients are properly selected, and surgery is planned and conducted thoughtfully. Furthermore, the ability of surgeons and designers to come up with the solution for each particular case in a collaboration, results in increased outcomes for patients and their quality of life [20]. In the case study, the patient is currently on a regular long-term follow-up after surgery and is satisfied with the outcomes.

## Conclusions

Fibrous dysplasia is a benign bone disorder of rare occurrence that warrants the attention of multiple medical specialties involved to diagnose and manage it properly. Developing favorable results and related improved patient's quality of life like the one in this case report are only possible with timely treatment and taking long-term care of the patients. However, the increased need for research is largely stipulated by an overall insufficient understanding of fibrous dysplasia pathogenesis and the extent of development of more appropriate treatment approaches for this hard-to-treat disorder.
